# Clinical Lectures on Diseases of the Eye

**Published:** 1864-06

**Authors:** E. L. Holmes

**Affiliations:** Chicago, Lecturer on Diseases of the Eye and Ear in Rush Medical College, and Surgeon of the Chicago Charitable Eye and Ear Infirmary


### CLINICAL LECTURES ON DISEASES OF THE EYE.

By E. L. HOLMES, M. D., of Chicago,

*Lecturer on Diseases of the Eye and Ear in Rush Medical College, and Surgeon of the Chicago Charitable Eye and Ear Infirmary.*

### THE CORNEA.—CHRONIC INFLAMMATION.

*Gentlemen :*—Under the term, Chronic Keratitis, I shall include the following forms of disease: vascular corneitis, phlyctenular corneitis, inflammation of the substance of the cornea, corneitis punctata, and shall close with a few remarks upon the sequelæ of corneitis, ulcers and opacities of the cornea.

These divisions will embrace nearly every form of chronic corneal inflammation, which you will most often meet in practice. You must not infer, however, that each class in all cases is characterized by well-marked, distinct symptoms. If you understand typical cases, you will readily comprehend such complicated cases as present many symptoms, common to several of the above-mentioned classes. I have termed these diseases chronic, since such is their usual character, although I should state that some of them occasionally terminate quite speedily in health. For the pathology of the different forms of Keratitis I must refer you to a former lecture.

1st. Vascular corneitis is usually a secondary disease, dependent upon long-continued irritation. This irritation may be produced by granulations upon the lids, rubbing over the cornea, by the presence of ulcers and phlyctænulæ of the cornea, by the action of foreign bodies, long retained under the lids, and by the constant exposure of the cornea to the air, as in ptosis and deficiencies of the lids from wounds, which prevent closure of the lids. One of the most favorable opportunities for studying the formation of vessels on the cornea is afforded by chronic conjunctivitis, especially when attended with granulations. At first the fine vessels are only observed, radiating over the limbus conjunctivæ. As the irritation continues, the inflammatory action invades the cells of the cornea, as shown by the presence of thin, nebulous patches upon the cornea. Accompanying this haziness the vessels are observed to advance beyond the limbus of the conjunctiva, upon the cornea. Ulcers of the cornea and phlyctænulæ very often render the surface of the cornea vascular; in these conditions, the vessels usually extend in small pencils from the periphery of the cornea to the points primarily diseased. Extreme vascularity of the cornea, with partially organized lymph, covering the cornea and somewhat resembling the granulations of a wound is termed pannus.

I shall say but little regarding the treatment of vascular corneitis. In a large proportion of cases, the vascularity disappears when the primary disease has been relieved.

2d. Phlyctænular corneitis is characterized by the presence of small inflamed elevated points, situated upon the cornea or at the union of the cornea and sclerotic. At first they are minute greyish points, which soon enlarge and become red, with increased vascularity of the surrounding tissues. Neuralgic pains, photophobia, lachrymation are frequent symptoms, variable however in violence. In mild cases there are few phlyctænulæ, and the irritation is confined principally to the globe. But very often there is intense pain, and swelling of the lids, with photophobia, so intense, that great distress is produced by the smallest amount of light that can enter the eye, when the patient is in a darkened room with the eyes bandaged. To obtain a satisfactory examination of the cornea it is necessary to place the patient under the influence of chloroform. This disease, or different phases of it, is known also as pustular ophthalmia, herpes cornea, scrofulous ophthalmia, and is principally found among children who suffer from a scrofulous diathesis.

I can only call your attention to a few of the general principles in the therapeutics of this disease. In treating this affection it is of primary importance for you to remember that the local disease is usually but a manifestation of constitutional derangement. It will be your duty, therefore, to improve the general health by means of necessary hygienic regulations, and by the use of such remedies as will correct the diathesis, as cod liver oil, iron, iodine, and occasionally bichloride of mercury in minute doses. Not unfrequently, I have found quinine, as recommended by Mackenzie and others, of great benefit, even when the patient is aparently not suffering from malarial influence. It is of the utmost importance to prevent patients from bandaging the eyes, and from burying the face in pillows and cushions, which such patients almost invariably do, when there is much photophobia. This causes congestion and prevents recovery.

The use of stimulating astringents requires much care. Trial of these remedies should be made, increasing or diminishing their strength as the case is found to require. I usually apply the solution of nitrate of silver, if found necessary, as I

described in the treatment of acute conjunctivitis, commencing with the strength of ten grains to the ounce. A very useful remedy in many cases is the yellow oxide of mercury, united with the glycerole of starch. An account of this remedy can be found in the first volume of the *American Journal of Ophthalmology.* With the best of treatment the disease is very liable to continue for a long time, or, if relieved, to return on exposure to changes of temperature.

3d. Inflammations of the deeper portions of the cornea are not uncommon. The first symptoms are, a loss of lustre and transparency of the cornea, the cloudy appearance being modified by the color of the iris. The disease may affect the whole cornea, or be confined to a portion of it. In proportion to its severity there will be redness of the conjunctivæ, photophobia, pain, and inability to keep the eye open. The cornea, or the affected portions of it, may become so opaque as to conceal the iris and pupil. At any stage in its course the disease may assume an acute form and speedily result in abscess or ulcer, or the same result may occur by a slow process.

A particular form of this disease, often complicated with iritis, and accompanying a peculiar development of the teeth, their edges being notched, as symptoms of inherited syphilis, has been described by Mr. Hutchinson, in a monograph worthy your perusal.

The treatment must be conducted in accordance with the principles already explained in connection with the other forms of corneal disease. Attention to the general health and mild local stimulants cautiously used in the form of weak solutions of arg. nit., vin. opii, and powdered calomel blown into the eye, are the chief remedies. Where pain is a prominent symptom, morphia internally, and atropia locally, are demanded.

4th. Corneitis punctata, would perhaps scarcely merit mention as a distinct disease, were it not so regarded in most works on diseases of the eye. It is characterized by numbers of fine grey points upon the cornea. Upon a careful examination the cornea is observed to be more or less covered with minute depressions as if made with the point of a needle. There is generally little pain or photophobia, but marked indistinctness of vision. The iris especially, and sometimes the choroid, retina and lens, are liable to suffer with the cornea. The disease almost invariably depends upon scrofulous or syphilitic diathesis, and is exceedingly obstinate. Constitutional treatment alone seems to be attended with benefit.

5th. Chronic ulcers of the cornea are chiefly observed in two forms, one accompanying granulated lids and vascular corneitis, and the other almost entirely without congestion of the eye. The first form often succeeds inflammation of the conjunctiva; the second, as a result of a minute point of corneal inflammation, precedes the vascular condition of the cornea and conjunctiva, which is simply a result, when it occurs, of the constant irritation produced by the presence of the ulcer. The first form will generally disappear if the primary disease is successfully treated. The second, like the other diseases above mentioned, require careful attention to the general health, and to a wise use of local stimulants. Although some of these ulcers are accompanied with scarcely any pain and congestion, they may resist all treatment for weeks. Preparations of lead should never be used locally in cases of ulcer of the cornea.

6th. I shall say but little regarding opacities of the cornea. You are aware that the greyish opaque portions of the cornea so often mentioned above, are manifestations of *existing* inflammation. The so-called opacities of the cornea are the result of past loss of the tissue; they are, in fact, scars, composed of newly-organized, but not transparent, tissue, thrown out to repair the loss. One of the chief diagnostic marks, distinguishing the two forms of opacity, is the aspect of their surfaces. In the first, the surface is usually dull and without lustre. In the second form the cornea has more or less recovered its brilliancy, though not its transparency. If the opacity is small and situated near the periphery of the cornea, vision is little obstructed. But if it is in front of the pupil, the usefulness of the eye is, to a greater or less extent, impaired. These opacities, if deep, can seldom be removed. The most successful treatment in cases of superficial opacities is gentle stimulation, as obtained by weak solutions of arg. nit., zinc, copper, and by electricity. The removal of these opacities with the knife is rarely warrantable, except in cases where, from the improper use of plumbi acetas, the carbonate of lead has become incorporated in the substance of a superficial cicatrix. The operation for artificial pupil, in cases of central opacity, will be described hereafter. It is a fact worthy of notice, that patients with central nebulous opacities, can often see much better through a minute aperture in a thin plate of metal than with the naked eye, since the diffraction of light is in a measure overcome.

